# The Effects of Ex Vivo Administration of Granulocyte-Macrophage Colony-Stimulating Factor and Endotoxin on Cytokine Release of Whole Blood Are Determined by Priming Conditions

**DOI:** 10.1155/2017/9834512

**Published:** 2017-12-14

**Authors:** A. Nierhaus, J. Linssen, M. S. Winkler, D. P. Frings, S. Kluge

**Affiliations:** ^1^Department of Intensive Care Medicine, University Medical Centre Hamburg-Eppendorf, Hamburg, Germany; ^2^Sysmex Europe, Norderstedt, Germany; ^3^Department of Anesthesiology, University Medical Centre Hamburg-Eppendorf, Hamburg, Germany

## Abstract

**Background:**

Lipopolysaccharide- (LPS-) induced tumour necrosis factor alpha (TNF*α*) secretion in critically ill patients can be considered as a measure of immune responsiveness. It can be enhanced by granulocyte-macrophage colony stimulating factor (GM-CSF). We investigated the effect of GM-CSF on ex vivo stimulated cytokine production using various preincubation regimens in healthy donors and patients with sepsis.

**Results:**

The maxima for the stimuli occurred 3 hours after stimulation. In donors, there was an increase (*p* < 0.001) of LPS-induced TNF*α* levels following incubation with GM-CSF. The* simultaneous* incubation with GM-CSF and LPS caused an inhibition of TNF*α* production (*p* < 0.001).* Postincubation* with GM-CSF did not yield any difference. In patients, preincubation with GM-CSF yielded an enhanced ex vivo TNF*α*-response when TNF*α* levels were low. Patients with increased TNF*α* concentrations did not show a GM-CSF stimulation effect. The GM-CSF preincubation yielded an increase of IL-8 production in patients and donors.

**Conclusions:**

This study demonstrates the immune-modulating properties of GM-CSF depending on the absence or presence of LPS or systemic TNF*α*. The timing of GM-CSF administration may be relevant for the modulation of the immune system in sepsis. The lack of stimulation in patients with high TNF*α* may represent endotoxin tolerance.

## 1. Background 

Trauma, haemorrhage, burns, pancreatitis, and severe infections may cause systemic inflammation [1–3]. An overwhelming and sustained proinflammatory immune response resulting in excessive levels of highly potent proinflammatory cytokines such as tumour necrosis factor alpha (TNF*α*), interleukin-1 (IL-1), interleukin-6 (IL-6), and interleukin-8 (IL-8) can lead to rapid development of shock and multiple organ failure (MOF) [4]. On the other hand, a predominantly anti-inflammatory reaction (termed compensatory anti-inflammatory response syndrome, CARS) induces immunosuppression with impaired host defence against pathogens [5, 6]. Such cellular immunoparalysis can be detected by a marked decrease of ex vivo whole blood lipopolysaccharide- (LPS-) induced TNF*α* and IL-8 production [7–9] which correlates with a decreased expression of Human Leukocyte Antigen-DR (HLA-DR), the essential antigen-presenting peptide receptor on monocytes [10, 11].

The intensity of the proinflammatory reaction was formerly considered to be a major determinant of clinical outcome during the initial phase. However, in recent years, the anti-inflammatory counterregulatory response has become the focus for research. Overall, the susceptibility and capability of an adequate response to infectious pathogens greatly influence patients' outcome [12–16].

Granulocyte-macrophage colony stimulating factor (GM-CSF) plays a key role in the endogenous response to infection and inflammation and has also been used in clinical experiments. GM-CSF, a 22 kDa glycoprotein cytokine, belongs to a group of growth factors (colony stimulating factors) promoting survival, clonal expansion, and differentiation of haematopoietic progenitor cells. GM-CSF induces committed progenitor cells (such as lymphoid and myeloid precursor cells) to proliferate and differentiate towards the granulocyte-macrophage pathways [17, 18]. In addition, GM-CSF modulates cell function [19] by enhancing the oxidative burst of neutrophils, eosinophils, and monocytes [20, 21], inducing a systemic release of proinflammatory cytokines such as IL-8 from neutrophils in vivo [22] and in vitro [23], inhibiting apoptosis [24–26], and promoting the expression of major histocompatibility complex class II molecules (HLA-DR) on monocytes* in vivo* [18, 27] and* in vitro* [28–30].

To understand the effects of GM-CSF on leukocytes* in vitro*, it is essential to create an environment closely resembling the* in vivo* situation in terms of circulating endotoxin levels. To investigate the potential suitability of GM-CSF as a therapeutic agent for the enhancement of innate immunity, we performed whole blood experiments using therapeutic concentrations of GM-CSF and similar endotoxin concentrations to those occurring in human septic shock [31, 32], while keeping exposure to stimuli as short as possible in order to avoid anergy of monocytes and neutrophils (no recruitment of new functional monocytes and neutrophils* in vitro*). Three different priming conditions were chosen to mimic the clinical situation to assess the pro- or anti-inflammatory properties of GM-CSF.

## 2. Materials and Methods

### 2.1. Healthy Volunteers and Patients

Following approval of the local research ethics committee (Hamburg State Chamber of Physicians, PV 1463), whole blood from 40 healthy donors (age: 16–72 years; median: 54 years) was used to determine the optimal concentration and incubation time for the activators lipopolysaccharide (LPS) and N-formylmethionyl-leucyl-phenylalanine (fMLP) in vitro. For GM-CSF stimulation, whole blood was used from another 28 healthy donors (age: 36–65 years; median: 51 years), as well as whole blood from 12 ICU patients with sepsis, 6 with an HLA-DR expression of <150 MFI and an ex vivo stimulation test of whole blood yielding a TNF*α*-response of <175 pg/mL (Millenium test, DPC Biermann), and another 6 with an HLA-DR expression of >150 MFI and an ex vivo stimulation test yielding a TNF*α*-response of >175 pg/mL. Whole blood and mononuclear cells were activated ex vivo with LPS or fMLP in different orders of priming. Informed consent was obtained from patients or their legal representatives.

### 2.2. Blood Sampling

EDTA blood samples (Sarstedt GmbH, Nümbrecht, Germany) were collected, processed, and incubated within 6 hours of collection.

### 2.3. Whole Blood Stimulation to Determine Optimal LPS and fMLP Concentrations and Incubation Time

We diluted 500 *μ*L of whole blood 1 : 1 with 500 *μ*L LPS to obtain concentrations of 50 ng/mL, 5 ng/mL, 500 pg/mL, 50 pg/mL, and 5 pg/mL, or with 500 *μ*L fMLP to obtain concentrations of 500 ng/mL, 50 ng/mL, 500 pg/mL, and 50 pg/mL. Incubation was timed stepwise starting with 1 min up to 6 hours. All dilutions were prepared in duplicate and incubated at 37°C. LPS and fMLP were obtained from* Escherichia coli* O111:B4 (Sigma GmbH, Deisenhofen, Germany).

### 2.4. Whole Blood from Healthy Volunteers and ICU Patients: Stimulation and Incubation with GM-CSF (Preincubation Modes A–C)

Preincubation with GM-CSF* (Leukine®, Sargramostim, Genzyme):*

- 500 *μ*L of whole blood was spiked with 250 *μ*L GM-CSF (5 ng/mL) and incubated for 3 hours in sterile pyrogen-free reaction tubes (Eppendorf GmbH, Hamburg, Germany). Thereafter, samples were incubated for 3 hours with 250 *μ*L LPS (end concentration: 500 pg/mL) or fMLP (end concentration: 50 ng/mL).
- Simultaneous incubation with GM-CSF and LPS/fMLP: 500 *μ*L of whole blood was diluted with 250 *μ*L GM-CSF (end concentration: 5 ng/mL) and 250 *μ*L LPS (end concentration: 500 pg/mL) or fMLP (end concentration: 50 ng/mL). This was followed by a 6-hour incubation period in sterile pyrogen-free reaction tubes.
- Preincubation with LPS or fMLP: 500 *μ*L of whole blood was primed with 250 *μ*L LPS (end concentration: 500 pg/mL) or 250 *μ*l fMLP (end concentration: 50 ng/mL) and incubated for 3 hours in sterile reaction tubes. Thereafter, samples were stimulated with 250 *μ*L GM-CSF (end concentration: 5 ng/mL) for 3 hours.

Stimulation regimens A–C were measured against controls incubated with normal saline. All dilutions were performed in duplicate and incubated at 37°C.

### 2.5. Isolation of Mononuclear (MN) Cells and Polymorphonuclear (PMN) Cells

Mononuclear (MN) and polymorphonuclear (PMN) cells were collected from healthy adult donors using dextran sedimentation and centrifugation with Ficoll-hypaque. The PMN fraction contained > 98% neutrophils and eosinophils. The MN fraction contained > 97% monocytes + lymphocytes. Lymphocytes were purified further from MN cells by centrifugal elutriation so that the final sample contained > 97% lymphocytes. All cell fractions were resuspended and diluted in their own plasma so that their concentrations were basically identical (CV < 5%) with whole blood conditions. Cell differentiation and cell counts were performed with a Sysmex XE-2100 haematology analyser (Sysmex Corp., Kobe, Japan).

### 2.6. Cytokine Measurements

Following the 3 h incubation period, the reaction tubes were centrifuged at 2°C and the concentrations of TNF*α* and IL-8 in the supernatant were measured with a commercially available automated system (Immulite®, Siemens/DPC Biermann, Bad Nauheim, Germany). The lower limits of detection of this system are 8 pg/mL for TNF*α* and 5 pg/mL for IL-8.

### 2.7. Monocyte HLA-DR Analysis by Flow Cytometry

Leukocyte phenotyping was conducted by dual-colour flow cytometry using a whole blood lysis technique and monoclonal antibodies (using phycoerythrin-conjugated CD14, fluorescein isothiocyanate-coupled CD45, and fluorescein isothiocyanate-coupled HLA-DR (all from Becton Dickinson, Heidelberg, Germany)). For technical details, see [27]. In short, diluted heparinized blood containing 5000–10,000 leukocytes/*μ*L was added to 20 *μ*L of antibody pairs for a final volume of 300 *μ*L and incubated in the dark for 15 min. Erythrocytes were lysed with lysing solution (Becton Dickinson, Heidelberg, Germany) and washed once with phosphate-buffered saline. Measurement of stained cells was performed on a FACSCanto (Becton Dickinson, Heidelberg, Germany). Monocytes were defined using scatter characteristics and CD14/CD45 staining. The monocyte population was analysed for HLA-DR expression which was expressed as mean fluorescence intensity (MFI).

### 2.8. Statistical Analysis

Statistical calculations were carried out using MedCalc®, Version 13.2 (MedCalc Software, Ostend, Belgium). More than two dependent subject groups were analysed using the Friedman test, followed by Bonferroni's correction for multiple comparisons. The paired Wilcoxon's test was used for pairwise comparisons between subject groups. The Mann–Whitney* U* test was used to analyse independent variables between groups. Values are expressed as mean plus SEM or SD, as indicated in the figure legends. Differences were considered significant at a *p* value of <0.05.

## 3. Results

### 3.1. Determination of Optimal LPS and fMLP Concentrations and Optimal Incubation Time for Whole Blood Stimulation

**Figure 1:**
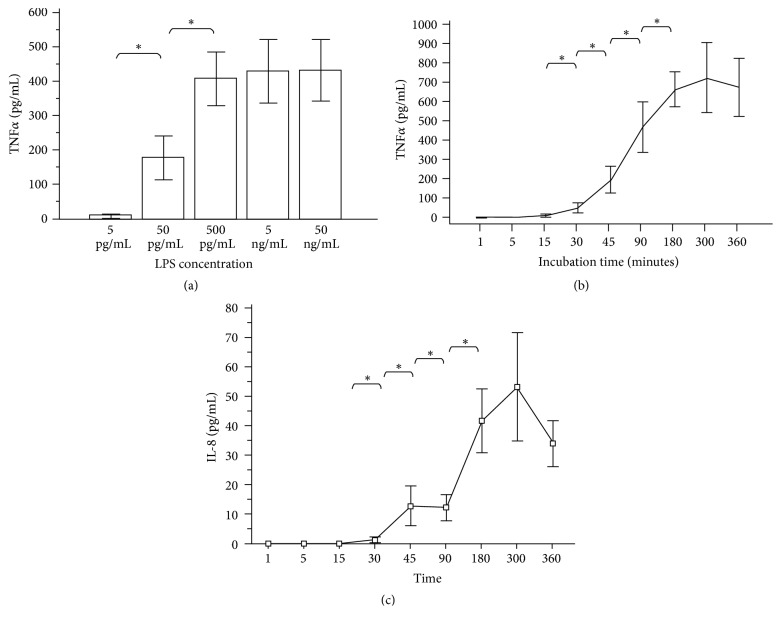
(a) TNF*α* production in whole blood from 40 healthy volunteers after 3 hours of incubation with different LPS concentrations. Data are presented as means ± SEM. ^*∗*^*p* < 0.05 = significant increase versus preceding concentration. (b) TNF*α* production over time in whole blood from 40 healthy volunteers after incubation with 500 pg/ml LPS. Data are presented as means ± SEM. ^*∗*^*p* < 0.05 = significant increase versus preceding concentration. (c) IL-8 production over time in whole blood from 40 healthy volunteers after incubation with 50 ng/mL fMLP. Data are presented as means ± SEM. ^*∗*^*p* < 0.05 = significant increase versus preceding concentration.

Figure 1(a) shows TNF*α* concentrations after 3 hours of incubation with increasing LPS concentrations (5 pg/mL–50 ng/mL) in blood from 40 healthy donors. The maximum concentration of TNF*α* production was reached at 500 pg/mL LPS with no further significant increase at higher LPS concentrations.  Figure 1(b) shows TNF*α* production over time induced by LPS (500 pg/mL) in blood from 40 healthy donors. The response started after 30 min incubation and reached its maximum after 3 hours without significant further increase at 6 hours. The production of IL-8, when induced by the chemotactic agent fMLP IL-8 (with a maximal response at a concentration of 50 ng fMLP/mL), showed an identical pattern with a maximum after 3 hours as illustrated in Figure 1(c). Both curves show an increase of the mean values at 300 min.

### 3.2. Healthy Volunteers: Ex Vivo GM-CSF Stimulation Modulates LPS-Induced TNF*α* and IL-8 Production and fMLP-Induced IL-8 Production in Whole Blood

**Figure 2:**
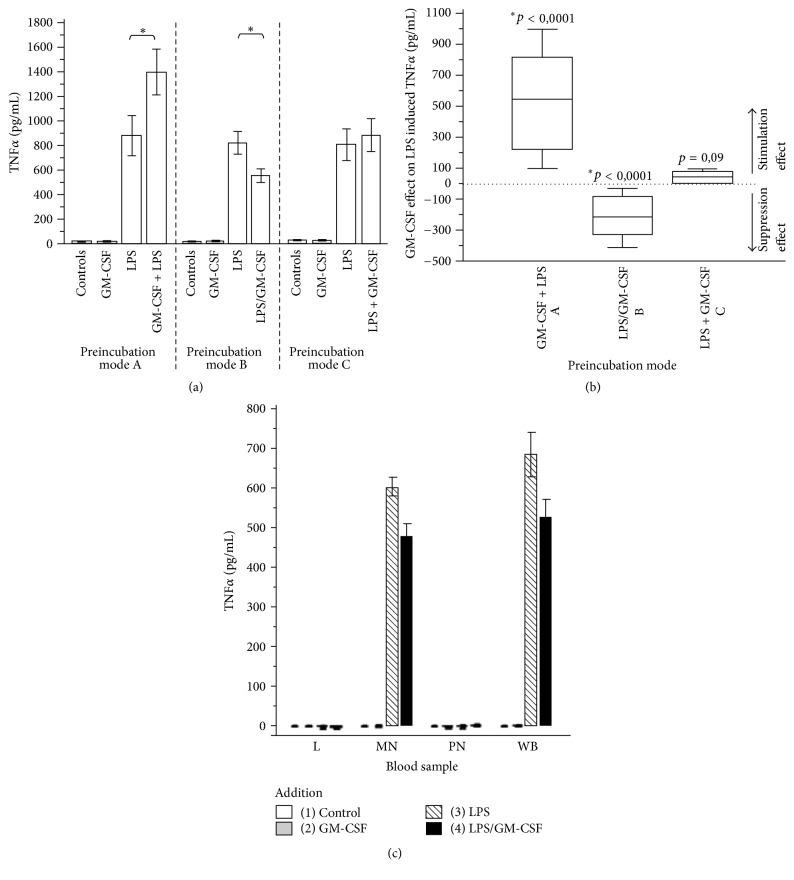
(a) LPS-induced (500 pg/ml) TNF*α* production in whole blood from 28 healthy volunteers under three different GM-CSF (5 ng/ml) preincubation conditions. Controls = no stimulation. GM-CSF = stimulation with GM-CSF only. LPS = stimulation with LPS only. Preincubation mode A: initial GM-CSF preincubation followed by LPS stimulation. Preincubation mode B: simultaneous stimulation of LPS and GM-CSF. Preincubation mode C: initial LPS preincubation followed by GM-CSF stimulation. Data are presented as means ± SEM. ^*∗*^Statistical difference with *p* < 0.05 = significant for GM-CSF effect on LPS-induced TNF*α* production versus stimulation with LPS only. (b) Box-and-whisker plot of the GM-CSF effects on LPS-induced TNF*α* production under three different preincubation conditions. The dotted zero line represents the absence of differences between LPS-induced TNF*α* production with and without GM-CSF administration. Preincubation mode A: initial GM-CSF preincubation followed by LPS stimulation. Preincubation mode B: simultaneous stimulation of LPS and GM-CSF. Preincubation mode C: initial LPS preincubation followed by GM-CSF stimulation. Data are presented as means ± SEM. ^*∗*^*p* < 0.05 = significant difference for GM-CSF effect on LPS-induced TNF*α* production versus no GM-CSF administration. (c) Stimulated TNF*α* production in whole blood (WB) compared to leukocyte subpopulations (L = lymphocytes, MN = monocytes + lymphocytes, and PMN = neutrophils + eosinophils). Data are presented as means ± SEM.

Incubating whole blood from healthy human donors with LPS (500 pg/mL) resulted in a massive release of TNF*α* and IL-8. When GM-CSF (5 ng/mL) was added following three different preincubation modes, the LPS-induced TNF*α* production was characteristically modulated (Figure 2(a)). A 3-hour preincubation with GM-CSF (5 ng/mL) followed by LPS stimulation (500 pg/mL; mode A) was followed by a significant increase of LPS-induced TNF*α* production. In contrast, the* simultaneous* application of GM-CSF (5 ng/mL) and LPS (500 pg/mL; mode B) resulted in a significant inhibition of TNF*α* production. GM-CSF stimulation (5 ng/mL) following incubation with LPS (500 pg/mL; mode C) did not show significant differences compared to control.  Figure 2(b) shows the GM-CSF net effect on TNF*α* release of the three different preincubation modes. The effect is given as (LPS + GM-CSF induced TNF*α* end concentration [pg/mL]) − (LPS-induced TNF*α* end concentration [pg/mL]).

GM-CSF alone did not enhance TNF*α* production in whole blood in the absence of LPS (+ normal saline: 19.6 ± 2.8 pg/ml TNF*α*; + GM-CSF: 21.5 ± 5.2 pg/ml TNF*α*). LPS stimulation or simultaneous LPS- and GM-CSF stimulation did not induce TNF*α* production in lymphocytes or PMN (neutrophils + eosinophils) after 6 hours of incubation with LPS (500 pg/ml) and/or 5 ng/mL GM-CSF. As expected, the induced TNF*α* production in whole blood was generated almost exclusively by monocytes (Figure 2(c)).

### 3.3. GM-CSF Modulates fMLP- or LPS-Induced IL-8 Production

**Figure 3:**
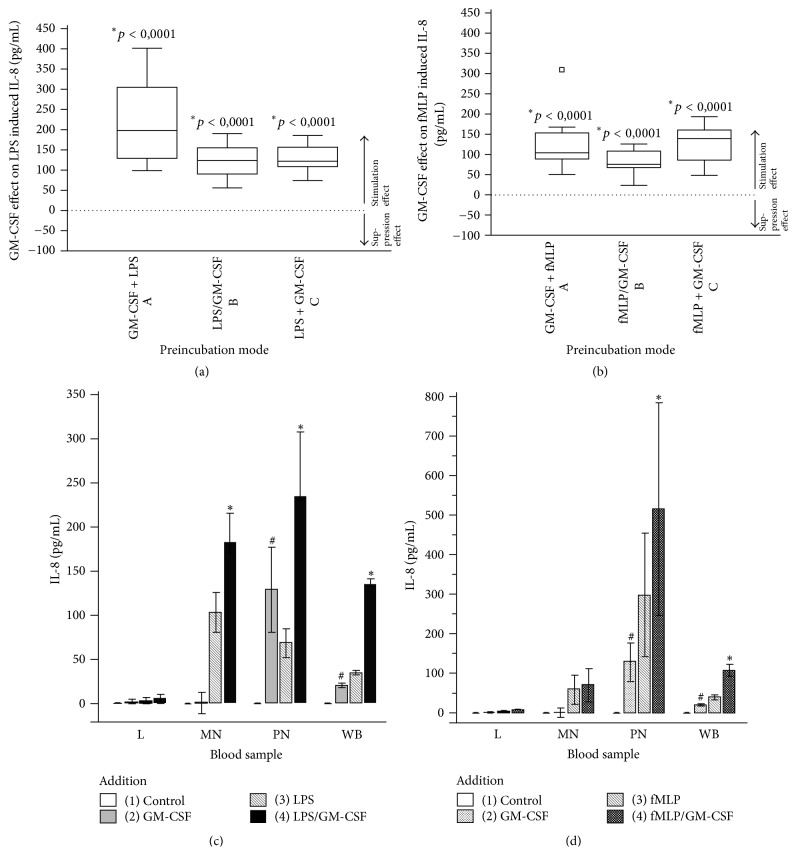
(a and b) Box-and-whisker plot of the GM-CSF effect on LPS-induced (a) and fMLP-induced (b) IL-8 production using three different preincubation modes. The dotted zero line represents the absence of differences between LPS- or fMLP-induced IL-8 production with and without GM-CSF. For the different incubation modes, compare Figures 2(a) and 2(b). ^*∗*^*p* < 0.05 = significant difference for GM-CSF effect on LPS- or fMLP-induced IL-8 production versus no GM-CSF administration. (c and d) LPS (c) and fMLP (d) plus GM-CSF induced IL-8 production in whole blood (WB) and leukocyte subpopulations (L = lymphocytes, MN = monocytes + lymphocytes, and PMN = neutrophils + eosinophils). Data are presented as means ± SEM. ^#^*p* < 0.05 = significant difference for IL-8 increase following GM-CSF (2) compared to controls (1). ^*∗*^*p* < 0.05 = significant difference for IL-8 increase following GM-CSF on LPS- or fMLP-induced IL-8 production (4) versus no GM-CSF administration (3).

The modulating effect of GM-CSF in fMLP- or LPS-induced IL-8 production was different from that for TNF*α*. Regardless of the type of “priming mode,” we consistently observed a significant increase in cellular IL-8 production (Figures 3(a) and 3(b)). Again, the net effect is given as (LPS or fMLP + GM-CSF induced IL-8 end concentration [pg/mL]) − (LPS- or fMLP-induced IL-8 end concentration [pg/mL]).

GM-CSF alone caused a significant IL-8 production in whole blood by the PMN fraction (neutrophils + eosinophils) in the absence of LPS or fMLP (IL-8 saline control ≤ 4 pg/mL and GM-CSF 30.5 pg/mL +/− SD 7.5) (Figures 3(c) and 3(d)). GM-CSF-induced IL-8 production was not observed in monocytes or lymphocytes. After initial LPS stimulation (Figure 3(c)), monocytes and PMN generated significant increases of GM-CSF-induced IL-8 in whole blood. This was different from fMLP stimulation (Figure 3(d)), where GM-CSF significantly increased IL-8 production only in PMN but not in monocytes. Lower IL-8 end concentrations in whole blood than in monocyte or PMN resuspensions are caused by IL-8 binding to the chemokine receptors on erythrocytes [22].

### 3.4. ICU Patients: In Vitro GM-CSF Stimulation Modulates LPS-Induced TNF*α*- and fMLP-Induced Cellular IL-8 Production in Whole Blood

We recruited 12 consecutive ICU patients with sepsis (Table 1) for this study. Six patients (of whom 2 had sepsis, 2 severe sepsis, and 2 septic shock) fulfilled the criteria for immunodepression according to locally established thresholds with critically decreased HLA-DR expression (<150 MFI) and ex vivo stimulated TNF*α* production (TNF*α* < 175 pg/mL; Millenium test, DPC Biermann, Bad Nauheim, Germany). Six patients had no immunodepression as defined above. Four of the 12 patients did not survive 28 days on the ICU, all of whom showed immunodepression.

Based on observations of variation in the GM-CSF effect on LPS- or fMLP-induced cellular TNF*α* or IL-8 production in whole blood from healthy volunteers, we studied the GM-CSF effect in a subgroup of patients with increased endogenous TNF*α* concentrations (in the absence of additional LPS or fMLP) and compared it with both the healthy volunteer group and a second patient group with normal endogenous TNF*α* levels. The endogenous TNF*α* and IL-8 concentrations of the ICU patients compared to the control group (*n* = 40) with a control mean TNF*α* of 19.6 pg/mL +/− 8.5 and IL-8 < 5 pg/mL showed that 5 out of 12 patients had increased endogenous TNF*α* levels of >30 pg/mL (32–45 pg/mL). Of these, two patients suffered from septic shock, both with immune-depression, one from sepsis (without immune-depression), one from severe sepsis (immunodepressed), and one patient from severe local soft tissue infection (hyperinflammatory).

**Figure 4:**
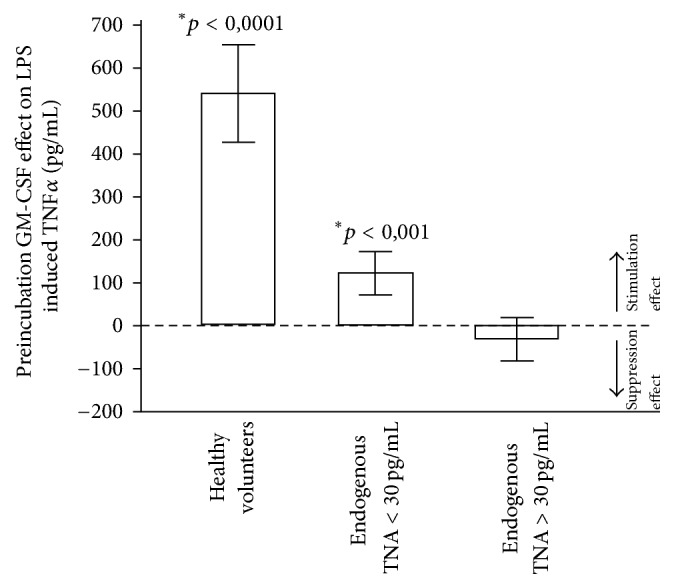
The effects of GM-CSF preincubation on LPS-induced TNF*α* production in blood from healthy donors (*n* = 40), patients with normal endogenous TNF*α* levels < 30 pg/mL (*n* = 7), and patients with increased endogenous TNF*α* levels > 30 pg/mL (*n* = 5). Data are presented as means ± SEM. ^*∗*^*p* < 0.05 = significant difference for TNF*α* increase following GM-CSF compared to TNF*α* release without GM-CSF administration.

The 3-hour preincubation with GM-CSF yielded a significant (*p* < 0.01) stimulation effect in the patients with low endogenous TNF*α* levels (<30 pg/mL). However, the effect was lower than in the control group of healthy volunteers. The patients with increased endogenous TNF*α* levels did not show a stimulation effect after GM-CSF on LPS-induced TNF*α* production (Figure 4).

**Figure 5:**
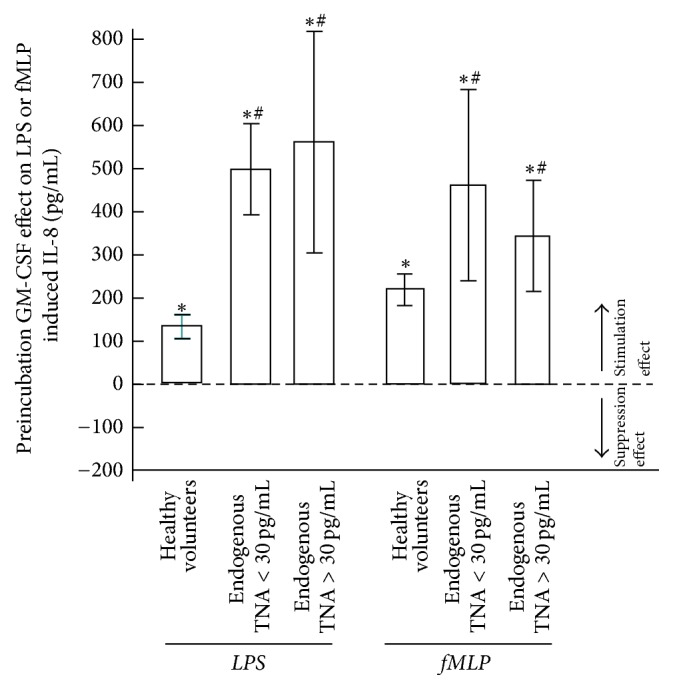
The effects of GM-CSF preincubation on LPS- and fMLP-induced IL-8 production in blood from healthy volunteers (*n* = 40), patients with normal endogenous TNF*α* levels < 30 pg/mL (*n* = 7), and patients with increased endogenous TNF*α* levels > 30 pg/mL (*n* = 5). Data are presented as means ± SEM. ^*∗*^*p* < 0.05 = significant difference for IL-8 increase following GM-CSF compared to IL-8 production without GM-CSF administration. ^#^*p* < 0.05 = significant difference for IL-8 compared to healthy volunteers.

Other than for TNF*α*, the GM-CSF preincubation effect on LPS- or fMLP-induced IL-8 production for all patients (with either normal or increased endogenous TNF*α*) showed a significant (*p* < 0.001) increase. Furthermore, we observed significantly higher overall GM-CSF stimulation effects on LPS-induced (*p* < 0.001) and fMLP-induced (*p* < 0.01) IL-8 production in the whole patient group than in healthy volunteers (controls) (Figure 5).

## 4. Discussion

Clinical studies have shown that an in vivo administration of GM-CSF can enhance the innate immune response by recruiting new functional monocytes and neutrophils. In neonates with sepsis and neutropenia, this led to increased total neutrophil counts and significantly decreased mortality [18]. In sepsis patients with ARDS, the administration of GM-CSF has been associated with an improvement in oxygenation [33]. GM-CSF restoring proinflammatory cytokine production capacity for TNF*α*, IL-8, and HLA-DR expression in patients with septic immunodepression is well documented [18, 21, 23, 27–30, 34]. Other in vivo GM-CSF trials have demonstrated the anti-inflammatory effect of growth factors (GM-CSF and G-CSF) by stimulating anti-inflammatory cytokine production such as IL-10, IL-1 receptor antagonist (IL-RA), and a TNF*α* antagonist, the soluble TNF receptor (sTNF receptors p55 and p75) [35, 36], all of which are known to effectively counterbalance an excessive immune response.

In vitro trials indicate that preincubation of blood samples with GM-CSF increases the LPS-induced cellular TNF*α* production and the HLA-DR expression on monocytes but also the secretion of anti-inflammatory cytokines such as IL-10, IL-RA, and sTNF receptor by monocytes in patients with severe sepsis [28, 29]. Interestingly, it has been found that whole blood from patients with severe MOF or patients with markedly increased endogenous TNF*α* levels could not be stimulated ex vivo by preincubation with GM-CSF [29]. There was also no therapeutic benefit when GM-CSF was given prophylactically to prevent infections following oncological surgery in a multicentre study [37]. In a preliminary study with immunodepressed severe sepsis patients [27], there were no differences in SOFA scores before and after GM-CSF administration. However, in a randomized, controlled, and biomarker-targeted trial, Meisel et al. demonstrated both the reversal of sepsis-associated immunosuppression and some clinical benefit in the GM-CSF treated group [34]. Such discrepant GM-CSF effects between in vitro and in vivo trials could possibly be explained by the generation of a novel monocyte and neutrophil population after in vivo treatment with GM-CSF, a phenomenon described in previous studies [19, 27]. The difference in effect between* in vitro* studies can be explained by the absence or presence of plasma components in isolated mononuclear cell cultures compared with that in reconstituted whole blood studies. Notably, the cytokine inhibiting capacity of plasma obtained from patients with sepsis and systemic inflammation has been shown previously [38]. Moreover, a long incubation time* in vitro* for GM-CSF and LPS or fMLP does not reflect in vivo settings since long-term GM-CSF will recruit new functional monocytes and neutrophils from the bone marrow and long-term in vitro GM-CSF incubation may increase the chance of endotoxin contamination.

Consequently, we investigated the ex vivo GM-CSF modulation effects in whole blood or in a subpopulation of isolated leucocytes resuspended in the original plasma of healthy volunteers and ICU patients with optimal concentrations of GM-CSF, LPS, and fMLP allowing the shortest possible incubation times. To reflect the GM-CSF modulation effect either with or without endotoxin or high endogenous TNF*α* levels, we studied the effect of different preincubation modes: initial preincubation with GM-CSF, simultaneous preincubation with GM-CSF and LPS/fMLP, or preincubation with LPS or fMLP.

In agreement with the ex vivo TNF*α* stimulation assay and the endotoxin levels in sepsis patients [31, 32], we found an almost identical optimal LPS concentration of 500 pg/mL after 3 hours for both patient groups. The optimum for fMLP was 50 ng/mL with an incubation time of 3 hours. When analysing the GM-CSF mediated effect in either the absence or presence of endotoxin, we found in healthy volunteers that GM-CSF alone at therapeutic concentrations of 5 ng/mL without LPS stimulation did not induce cellular TNF*α* production in whole blood or in novel monocyte populations. This was not in concordance with findings reported by Williams et al. [28]. One possible reason for such differences might be endotoxin contamination resulting from an incubation time exceeding 36 hours in their study. In agreement with previous reports, we found that preincubation with GM-CSF increased the* LPS-induced* TNF*α* production. The stimulating effects on cellular TNF*α* production and on HLA-DR expression (not investigated in this study) by “priming” with GM-CSF are well documented [19].

On the other hand, a simultaneous incubation with GM-CSF and LPS revealed a contrary effect compared to GM-CSF preincubation alone: LPS-induced cellular TNF*α* production was inhibited or suppressed. Nishiki et al. [39] reported similar results with* in vitro* application of G-CSF in a novel monocyte population. They showed that G-CSF inhibited both TNF*α* release and TNF*α* mRNA expression by activating the STAT3 pathway in the same way as IL-10. Our results show that GM-CSF effectively modulates an anti-inflammatory response when blood samples from healthy volunteers are primed simultaneously with GM-CSF* and* LPS in vitro. Finally, preincubation of the same samples with LPS did not yield any significant difference with or without GM-CSF when measuring the LPS-induced TNF*α* production. Such markedly reduced response to GM-CSF could well be in line with the decreased reactivity of monocytes in patients with septic shock or elevated endogenous TNF*α* concentrations (“endotoxin tolerance”). As a consequence, our results indicate that the timing of GM-CSF or G-CSF administration might be crucial for successful cytokine modulation in order to enhance the innate immune response of patients treated for sepsis [40].

For the fMLP- or LPS-induced IL-8 production, the modulating or “priming” effect of GM-CSF is different from TNF*α*. Independent from any priming regimen or stimulus, there was always a significant increase of IL-8 production in whole blood from healthy volunteers. Pretreatment with GM-CSF alone, in the absence of LPS or fMLP, led to significant IL-8 production in whole blood and in PMN (neutrophils + eosinophils) samples while such an effect was not induced in monocyte or lymphocyte resuspensions. On the other hand, the modulating effect of GM-CSF on LPS-induced IL-8 production differed from the one obtained for fMLP-induced IL-8 production. While the effect of GM-CSF on LPS-induced IL-8 end concentrations originated from monocytes and PMN activity, the fMLP effect resulted only from PMN and not from monocytes. Several studies [22–26] have shown that GM-CSF and IL-8 delay or inhibit the apoptosis of PMN and monocytes by activation of ERK and the PI-3-kinase pathway. Further, it had been shown that treatment of neutrophils with GM-CSF enhanced IL-8 secretion and superoxide generation in response to TLR2 ligands. GM-CSF enhancement of neutrophil responses was receptor-specific; that is, the response to TLR2 but not TLR4 ligands was dramatically increased in GM-CSF treated neutrophils [41].

Based on the observation that a modulating effect of GM-CSF on LPS- or fMLP-induced TNF*α* or IL-8 production in whole blood of healthy volunteers is greatly influenced by preincubation conditions, we studied the response to GM-CSF administration in our patient group with elevated constitutive TNF*α* plasma levels (in the absence of additional LPS or fMLP). Results were compared to those from healthy volunteers and to the patient group with normal TNF*α* levels. In patients with normal endogenous TNF*α* levels, a three-hour incubation with GM-CSF resulted in an enhanced cytokine response even though the effect was lower than that in the control group. In contrast, and in agreement with previous reports [29], the blood from patients with increased endogenous TNF*α* concentrations did not show any GM-CSF-related stimulation effect.

On the other hand, the GM-CSF preincubation led to an increase in LPS- or fMLP-induced IL-8 production in blood from both patients and healthy volunteers. IL-8 production following LPS or fMLP stimulation in patients with normal or elevated endogenous TNF*α* levels was significantly higher than that in healthy volunteers.

These findings indicate that GM-CSF may be of limited therapeutic use in the presence of elevated endogenous TNF*α* levels due to hyporesponsiveness of the innate immune function that has been blunted by LPS. At the same time, neutrophil IL-8 production was preserved if not enhanced.

Our study has several limitations. Due to the small number of patients, our conclusions cannot be extrapolated to the general patient population and need to be confirmed in a larger study. Also, it should be considered that the effects of a single dose of a growth factor in healthy volunteers may differ from the effects of repeated doses of the same growth factor in patients in different disease states. One of the final goals for successfully treating patients is to supply the biological system with sufficient quantities of functionally mature leukocytes allowing an adequate and timely normalization of the immunoinflammatory response [5]. Finally, we are aware of the fact that an ex vivo experiment only partially represents the complex* in vivo* environment in sepsis.

## 5. Conclusion

This ex vivo study demonstrates that the immunomodulating effects of the haematopoietic growth factor GM-CSF on circulating leukocytes in healthy volunteers and septic ICU patients depend on the presence or absence of elevated LPS or endogenous TNF*α* levels. Our findings suggest that the timing of GM-CSF administration may be relevant for obtaining an effective modulation of the cytokine response of circulating leukocytes in sepsis.

## Figures and Tables

**Table 1:** Patient characteristics and immune parameters on day 1.

Patients' diagnoses	SAPS II	Microbiology (pathogen species)	Mortality (28-day)	HLA-DR (MFI)	TNF*α* plasma concentration (pg/ml)	Ex vivo stimulated TNF*α* (pg/ml)	Ex vivo stimulated IL-8 (pg/ml)
Pneumonia	38	*Klebsiella*	S	825	18	917	1008
Esophagectomy	33	*E. coli*	S	300	19	362	242
Pneumonia	40	*S. aureus*	S	399	18	825	899
Multiple trauma	58	*E. coli*	S	375	27	342	177
Peritonitis	37	*Enterobacter*	S	312	20	206	294
Pancreatitis	59	*E. coli*	S	185	42	53	77
Peritonitis	32	*Enterobacter*	S	95	45	71	69
Peritonitis	51	*Klebsiella*	S	147	24	117	168
Pneumonia	60	*Hemophilus*	NS	23	33	34	55
Peritonitis	54	*Serratia*	NS	79	35	20	44
Peritonitis	58	*E. coli*	NS	105	32	68	125
Multiple trauma	63	*S. aureus*	NS	147	28	121	133

SAPSII: Simplified Acute Physiology Score II; HLA-DR: Human Leukocyte Antigen-DR; MFI: mean fluorescence intensity; TNF*α*: tumor necrosis factor alpha; IL-8: interleukin-8; S: survivor; NS: nonsurvivor.
